# Organic Solar Cells Parameters Extraction and Characterization Techniques

**DOI:** 10.3390/polym13193224

**Published:** 2021-09-23

**Authors:** Mahmoud N. Zidan, Nicola Everitt, Tawfik Ismail, Irene S. Fahim

**Affiliations:** 1 Smart Engineering Systems Research Center (SESC), Industrial Engineering Department, Nile University, Giza 12677, Egypt; m.nazih@nu.edu.eg; 2 Department of Mechanical, Faculty of Engineering, Materials and Manufacturing Engineering, University of Nottingham, Nottingham NG72RD, UK; nicola.everitt@nottingham.ac.uk; 3 National Institute of Laser Enhanced Sciences, Cairo University, Giza 12613, Egypt; tismail@cu.edu.eg; 4 Wireless Intelligent Networks Center (WINC), Nile University, Giza 12677, Egypt

**Keywords:** organic solar cells, parameter extraction, mobility, recombination, CELIV, SCLC, TOF

## Abstract

Organic photovoltaic research is continuing in order to improve the efficiency and stability of the products. Organic devices have recently demonstrated excellent efficiency, bringing them closer to the market. Understanding the relationship between the microscopic parameters of the device and the conditions under which it is prepared and operated is essential for improving performance at the device level. This review paper emphasizes the importance of the parameter extraction stage for organic solar cell investigations by offering various device models and extraction methodologies. In order to link qualitative experimental measurements to quantitative microscopic device parameters with a minimum number of experimental setups, parameter extraction is a valuable step. The number of experimental setups directly impacts the pace and cost of development. Several experimental and material processing procedures, including the use of additives, annealing, and polymer chain engineering, are discussed in terms of their impact on the parameters of organic solar cells. Various analytical, numerical, hybrid, and optimization methods were introduced for parameter extraction based on single, multiple diodes and drift-diffusion models. Their validity for organic devices was tested by extracting the parameters of some available devices from the literature.

## 1. Introduction

Organic solar cells have been under massive research for almost two decades to compete with silicon solar cells in power conversion efficiency and stability. High power conversion efficiency had been achieved by incorporation of non-fullerene and small molecule acceptors, which exhibit high absorption spectrum resulting in high photo-current [1,2]. Power conversion efficiency above 18% has been achieved in the state-of-the-art device [3,4,5]. Advantages of organic solar cells such as flexibility, low production cost, transparency, and simple roll-to-roll production techniques make it a promising technology for energy conversion that may take over silicon-based technologies in the future. It also can be integrated with some applications such as bendable and stretchable electronics [6,7], an integrated power source for sensors in the internet of things applications (IoT) [8] and a replacement of windows in buildings front for aesthetic appearance and power generation [9]. Although the technology has achieved relatively high power conversion efficiency for small area devices. It can not achieve this high efficiency with a large area or maintain it for a long time. Besides stability issues, there are some environmental concerns regarding this technology, such as the toxicity of used solvents. Currently, research is focusing on increasing the stability and optimization of the device for absorption enhancement, improved energy yield, and finding efficient and environment-friendly materials [10,11].

Polymer materials are included in different types of solar cells according to their optical and electrical properties. The use of polymer materials in different layers is proposed to improve the efficiency of solar cells. For example, polymers like polyethylene terephthalate (PET) and polyethylene naphthalate (PEN) are used as a substrate due to their high flexibility, and transparency [12]. An example of polymers as active layer materials are PVK-MEHPPV:PCBM [13], and P3HT:PCBM, which has been under massive research in the past decade [14], additive or dopant material for active and hole transport layers morphology modifications in different types of solar cells [15]. Polymers are used as an interfacial layer also. A widely used interfacial layer due to its high conductivity consists of two polymers mixed, commonly known as PEDOT:PSS [16].

As the photovoltaic effect in organic materials is more complicated than inorganic semiconductors, more understanding and investigation of device physics and behavior is required to achieve better performance. Several factors affect cell performance, such as carrier mobility, traps, charge carriers densities, recombination, and parasitic resistances. Various measurement and characterization techniques should be done to study the behavior of the solar cell thoroughly. This is considered a costly and time-consuming process. In such situations, researchers incorporate simulation tools and theoretical studies in their work. Some of these studies come before taking the device into the lab to get insights into the optimal fabrication parameters to start the experiment. Others consider the parameters of the fabricated device, especially for devices based on newly developed materials. Theoretical studies consider several aspects of the device, including device physics, structure, selection of materials, and operating conditions. Several models for describing the device physics based on models developed for silicon solar cells have been introducing alongside their modifications to accommodate the differences between organic and inorganic devices. Recently, machine learning models have been used in the field to speed up development. For example, machine learning models are used for selecting the best donor-acceptor combination and for predicting the cell properties [17,18,19,20].

One of the essential steps of the device design and improvements process is to extract its parameters to study the effect of each experimental condition or structure modification on the device physics for further enhancement, and optimization [21,22]. Most of the literature focuses on a single part of the parameters study. Either it represents theoretical methods for extracting the device parameters or studying a single parameter experimentally. Theoretical studies provide a mathematical derivation of the reported method with reporting on its accuracy compared to other studies without further interpretation of the extracted parameters and their relation to a specific experimental condition like temperature, the concentration of used solutions, materials, or device structure. The experimental section of the literature reports the effect of parameters of interest. In most cases, the findings were represented in terms of power conversion efficiency with few mentions of the microscopic parameters.

This article provides information about several models of organic solar cells and a set of experimental and material processing techniques used to tune active and interfacial layers materials. The article summarizes several analytical, numerical, and optimization methods for parameter extractions concerning each method’s assumptions. In this work, theoretical methods with common experimental techniques are combined to offer a comprehensive review for studying organic solar cells in light of progress made in the field of photovoltaics on the perspectives of materials enhancement and experimental measurement methods such as current-voltage measurements, charge extraction by increasing voltage (CELIV) and time of flight technique.

## 2. Models of Solar Cell

J−V curve is one of the most important device measurements that could be performed; it offers information about several device parameters such as open-circuit voltage, short circuit current density, fill factor, and maximum power point (MPP). The overall device performance can be determined from the curve. Although these parameters are the standard parameters to study the solar cell’s performance, they do not explain the effect of material preparation conditions on the device physics in a quantitative manner necessary for research and performance enhancement of state-of-the-art devices. Parameters such as series resistance and shunt resistance can be extracted from the J−V curve as the increase in the resistance will result in a lower fill factor making the shape of the J−V curve flatter [23,24]. Extraction of parasitic cell resistances is essential to study the solar cell and how different processing and preparation conditions affect its electrical parameters, especially in organic solar cells. The characteristic of solar cells can be described by several models [25,26,27,28,29,30]. Single and double diode models are widely used to study different types of the solar cell.

### 2.1. Single Diode Model

**Figure 1:**
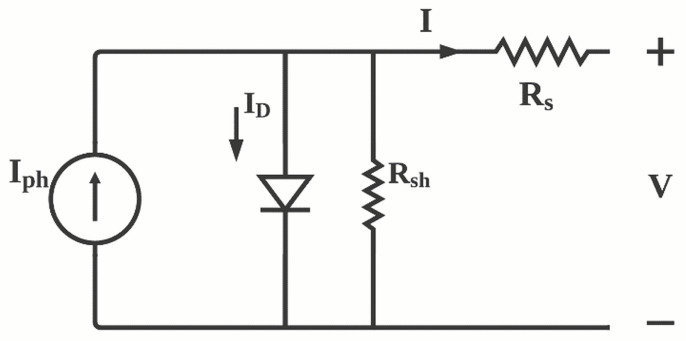
Single diode model of solar cell: *I* is the device current, *V* is the cell voltage, $K_{B}$ is the Boltzmann’s constant and *T* is the absolute temperature.

Due to its simplicity and it has been proved to be efficient for parameter extraction for different types of solar cells. Single diode model is commonly used to study photovoltaic devices. The single diode model shown in Figure 1 considers five parameters as a representation of the cell [31,32,33]. The photo-generated current $\left(I_{ph}\right)$, diode reverse saturation current $\left(I_{D}\right)$, ideality factor (n), series resistance $\left(R_{s}\right)$ and shunt resistance $\left(R_{sh}\right)$.

Cell parameters exhibit different relations with solar irradiance (G) and temperature. The accuracy of the assumed relation determines the error between the exact values of the devices and the extracted one. In single diode model, $\left(I_{ph}\right)$ is assumed to be linearly dependent on *G* therefore the cell current is linearly dependent on the solar irradiance. This assumption includes some inaccuracy and other nonlinear relations were introduced to improve the model. However, the model fits experimental data to an acceptable level of accuracy [34].

#### Parameters Extraction from Single Diode Model

The nonlinear five-point model depends on the knowledge of key points on the I−V curve to extract the parameters of interest. Most of the analytical methods in the literature use a subset or all parameters named, The open circuit voltage $\left(V_{oc}\right)$, short circuit current $\left(I_{sc}\right)$, voltage and current at the maximum power point $\left(V_{mpp},I_{mpp}\right)$, slope at short-circuit point $\left(R_{sh0}\right)$ and the slope at the open-circuit point $\left(R_{s0}\right)$. Modification to this method based on the observation that the dependency of parasitic resistances on solar irradiance showed a power law relation. The modified method incorporates three empirical parameters, α considers the nonlinear dependence of $I_{sc}$ on irradiance, β is a dimensionless coefficient specific to device technology and γ considers the non-linear dependence of the voltage on temperature. Accurate determination of these coefficients is essential to accurate extraction of $R_{s}$ and $R_{sh}$. The cell voltage and current must be determined at several operating points to get accurate values which complicates the process of parameters extraction although, the modified method results in better fitting for experimental I−V data which deserves the additional work [35]. By assuming high $R_{sh}$ approaching infinity, the model is simplified to a four-parameter model by neglecting the shunt resistance [36]. By using Lambert W function, an analytical form of cell current as a function of voltage is used to extract the cell ideality factor (n), series resistance $\left(R_{s}\right)$ and shunt resistance $\left(R_{sh}\right)$ [37,38,39]. The equation can be solved using different numerical methods of curve fitting such as Newton’s methods [40] and Levenberg-Marquard [41]. By applying the least square method to the Lambert W based model three parameters $\left(R_{sh},R_{s},n\right)$ can be extracted directly [42] and getting the other two by simple calculations. Although this method reduces the number of parameters to be extracted, it requires the knowledge of the whole I−V curve data points specially the points of $V_{oc}$ and $I_{sc}$ which may not be available in some measurements and an initial guess of the parameters of interest close to the real values is needed for the sake of convergence. For missing $V_{oc}$ and $I_{sc}$ data, extrapolation of the curve to get these points will extend the usability of this method. The method was applied successfully to extract the parameters of silicon cells, modules, and organic solar cells. Another analytical method developed by Celik and Acikgoz [43] extracts the five parameters by calculating $R_{s}$ and $R_{sh}$ from the derivative at and respectively as given by (1)–(4).

(1) $$\frac{dI}{dV}=\frac{\frac{-I_{0}}{n\;N_{s}\;T\;e^{\left(\frac{V_{oc}}{n\;N_{s}\;T}\right)}}+\frac{1}{R_{sh}}}{1+R_{s}\left(\frac{-I_{0}}{n\;N_{s}\;T\;e^{\left(\frac{V_{oc}}{n\;N_{s}\;T}\right)}}+\frac{1}{R_{sh}}\right)}-\frac{1}{R_{sh0}},$$

(2) $$R_{sh}=R_{sh0},$$

(3) $$R_{s}=R_{s0}-\left[n\;N_{s}\;\frac{K_{B}\;T}{q}\;exp\left(\frac{-V_{oc}}{n\;N_{s}\;\frac{K_{B}\;T}{q}}\right)\right],$$

(4) $$n=\frac{V_{mpp}+I_{mpp}\;R_{s0}-V_{oc}}{N_{s}\;\frac{K_{B}\;T}{q}\left[ln\left(I_{sc}-\frac{V_{mpp}}{R_{sh}}-I_{mpp}\right)-ln\left(I_{sc}-\frac{V_{oc}}{R_{sh}}\right)+\frac{I_{mpp}}{I_{sc}-\frac{V_{oc}}{R_{sh}}}\right]}$$

where $N_{s}$ is the number series connected cells in the module. For single cell parameter extraction $N_{s}$ = 1.

Another method which depends only on the key points of the I−V curve and avoids the need for open circuit conditions and maximum power point had been developed and represented in [44]. An analytical method that benefits from numerical integration to get the fifth equation of the five parameters model without assumptions or simplification to the model. The method calculates the area under the I−V curve and relates the result to the model parameters. The accuracy of this method depends on the accuracy of the numerical integration and the number of data points [45].

Several analytical representations of device parameters based on different assumptions to simplify the calculation had been introduced in the literature. For Example, in [43] the shunt resistance is assumed to infinite, so it is neglected. The photo-generated current is assumed to be equal to the short circuit current at the standard test conditions by neglecting the value of diode current compared to $I_{ph}$ [46]. Different approximations reducing the number of parameters from five to one were reported in the review papers in [47,48]. A full numerical method that makes use of Lambert W function to represent the voltage in terms of current is used to extract the five parameters by understanding the five points on the experimental I−V curve. Orthogonal distance regression is used to fit the model to experimental data with linear fitting at $V_{oc}$ and $I_{sc}$ to calculate the initial values for parasitic resistances [49]. Another method constructs five algebraic equations that represent the coefficients of a co-content function calculated from the analytical solution of the I−V equation [50]. The coefficients are represented in terms of device parameters which could be extracted by means of fitting. Improved adaptive differential evolution algorithm is used to extract the parameters with good accuracy and convergence speed compared to the original JADE algorithm [51].

One of the drawbacks of the single diode model is the resistances cannot be extracted accurately from S-shaped which is a common case when studying the effect of some experimental processes on organic solar cells, so modifications to the model had been done to overcome these limitations alongside different extraction methods. By use of double diode model, S-shaped J−V curve of an organic solar cell treated by oxygen plasma had been regenerated [52]. It was found that decreasing the surface recombination velocity of the majority carriers produces the deformation of the J−V curve. Beside the surface recombination velocities strong mobility imbalance and low mobilities in planar heterojunction solar cells [53] can cause an s-shaped I−V curve. It is important to relate the deviation from the ideal I−V curve to the physical conditions that produced such deviations and link them to the affected parameter on the I−V curve whether it is the *n*, $R_{s}$ or $R_{sh}$. Another limitation on the accuracy of single diode model, the assumption of $R_{sh}\gg R_{s}$ is valid for most of silicon solar cell but it is not the case in thin film solar cells and organic solar cells.

### 2.2. Double Diode Model

**Figure 2:**
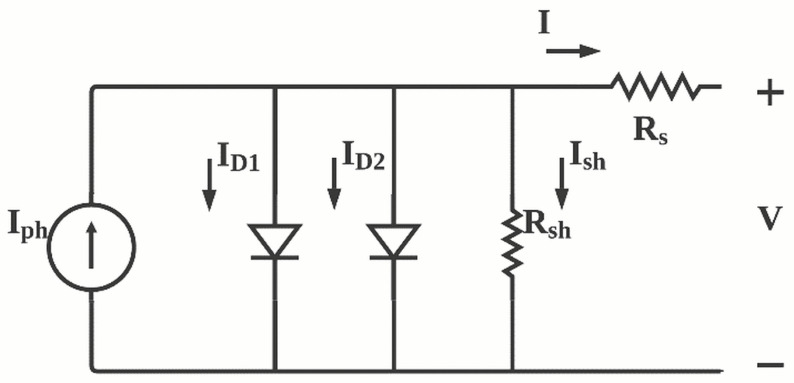
Double diode model of solar cell.

The double diode model represents a more accurate model of the photovoltaic devices. The double diode model is represented by Figure 2 and (5)–(7) [54,55,56]. The model incorporates two additional parameters $I_{D2}$ which represent the saturation current due to charge recombination in space charge region and $n_{2}$ as the ideality factor for generation and recombination currents.

(5) $$I=I_{ph}-I_{D1}-I_{D2}-I_{sh},$$

(6) $$I_{D1}=I_{SD1}\;\left(\;exp\left(\frac{V_{L}+I_{L}\;R_{s}}{n_{1}\;K_{B}\;\frac{T}{q}}\right)-1\right),$$

(7) $$I_{D2}=I_{SD2}\;\left(\;exp\left(\frac{V_{L}+I_{L}\;R_{s}}{n_{2}\;K_{B}\;\frac{T}{q}}\right)-1\right)$$

#### Methods for Parameter Extraction Using Double Diode Model

There are many analytical, numerical methods, and a combination has been developed to extract the model’s parameters. An analytical method based on the assumption of $n_{1}=1$ and $n_{2}=2$ had been made, reducing the number of parameters to five like in the single diode model. A hybrid method that depends on evaluation of the double diode model at $V_{oc},I_{sc}$ and MPP. The set of produced equations is then solved numerically using Newton’s Raphson method. The equations are further approximated for convergence consideration, and a new set of equations represents the diode saturation current introduced in [57].

Extraction of $n_{1}$ and $n_{2}$ based on the iterative fixed-point method for solving non-linear equations is represented in [58]. Genetic algorithm method is applied to the double diode model of the solar cell to improve the accuracy of the extracted parameters [59]. In this method, an initial estimation of cell parameters by another technique is needed. The GA technique resulted in worst-case error of 35% when the values are out of range by ±100%. Bouzidi introduced a method that fits the double diode model to experimental data by dividing the I−V curve into two regions with the assumption of $n_{1}=1$ and $n_{2}=2$ [60]. In this method, the shunt conductance $G_{sh}=\frac{1}{R_{sh}}$ is extracted instead of the shunt resistance. One portion of the curve accounts for voltage error at high voltages near the $V_{oc}$ and the other considers the error in the current at small voltages. An advantage of this method, it does not need an initial guess of the parameters for the least square algorithm to converge.

Extraction of the model parameters from dark I−V data by assuming constant $n_{1},n_{2}$ and $R_{s}$ was introduced in [61]. The least square method is used to fit the standard deviation of current. A method that makes use of analytical and numerical method was introduced in [62,63]. The numerical part is used to extract the values of the series resistance and the two ideality factors. The rest of the model parameters were calculated analytically by developing three analytical equations at the open circuit, short circuit and MPP points as introduced in the previous section of the single diode model. The Quadratic and cubic solution of $R_{s}$ were compared showing that the cubic solution is more accurate than the quadratic one due to the following approximation for simplicity purposes.

(8) $$exp\left(k\;R_{s}\right)=1+k\;R_{s},$$

where $k=\frac{I_{m}}{K_{B}\;\frac{T}{q}}$ or $k=\frac{I_{m}}{2\;K_{B}\;\frac{T}{q}}$.

An explicit double diode model is represented in terms of Lambert W function, containing seven parameters such as the conventional double diode model and improved teaching-learning optimization algorithm is used for parameter extraction [64].

### 2.3. Notes on the Conventional Models

**Figure 3:**
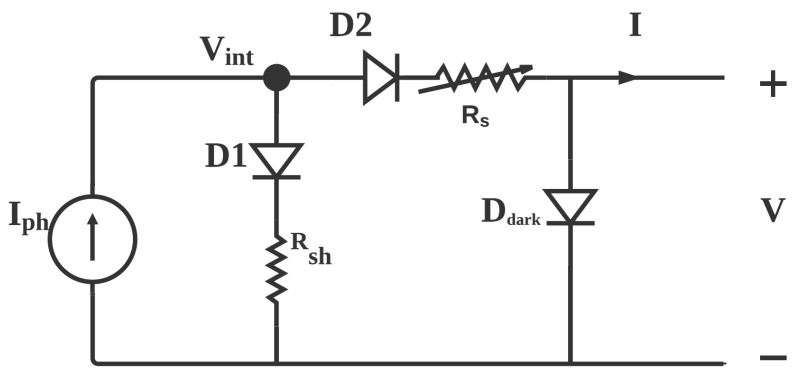
Proposed model for OPV by Mazhari.

Single and double diode models were based on some assumption which may not be valid for organic solar cells such as constant $I_{ph}$ so, modifications to these models were made to accurately represent the behavior of this technology. Mazhari, introduced a parameter $\zeta =\frac{f(V_{oc})}{I_{sc}}$ to estimate the validity of conventional models to represent the organic solar cells, where $f(V_{oc})$ represents the dark current characteristics of the device. The closer the value of ζ to 1, the conventional models are suitable to represent organic solar cells [65]. The author also suggests a modified model to represent the organic solar cells which accounts for recombination, extraction of charges and the dark I−V characteristics of the solar cell each represented by a diode to model the non-linear characteristics. The modified model is represented in Figure 3 with shunt resistance models the recombination, the series resistance models the loss of extraction of charge carrier. $R_{s}$ and $R_{s}h$ are intrinsic values not extrinsic as they are in the conventional models.

Extraction of shunt $R_{sh}$ is possible by finding a voltage at which the current is almost the same in dark and light conditions. $R_{s}$ can be calculated by knowledge of $R_{s}h$ and $I_{ph}$ from the following relation.

(9) $$R_{s}=\frac{\left[I_{ph}-I-f\left(V\right)\right]\;R_{sh}-V}{I_{ph}-I-f\left(V\right)}$$

In previous discussion we represented several methods for parameters extraction based on single and double diode models using different techniques. For more covering of the topic Table 1, lists additional methods for parameters extraction.

Another model that is widely used to study organic solar cells is the drift-diffusion model which is based on set of Equations (10)–(14) that includes the microscopic parameters of the device.

(10) $$\frac{d}{dx}\;\epsilon_{0}\;\epsilon_{r}\;\frac{d\phi }{dx}=q\;\left(n-p\right)$$

(11) $$\frac{\partial J_{n}}{\partial x}=q\;\left(R_{n}-G+\frac{\partial n}{\partial t}\right)$$

(12) $$\frac{\partial J_{p}}{\partial x}=-q\;\left(R_{p}-G+\frac{\partial p}{\partial t}\right)$$

(13) $$J_{n}=q\;\mu_{n}\;\frac{\partial E_{c}}{\partial x}+q\;D_{n}\frac{\partial n}{\partial x}$$

(14) $$J_{p}=q\;\mu_{p}\;\frac{\partial E_{v}}{\partial x}+q\;D_{p}\frac{\partial p}{\partial x}$$

where $\epsilon_{0},\epsilon_{r}$ are permittivity of the free space and the relative permittivity of the organic blend, ϕ is the voltage profile, *x* is the dimension perpendicular to cell surface, $J_{n}$ and $J_{p}$ are electron and hole current densities, *n* and *p* are electron and hole densities, $R_{n/p}$ is the recombination rate of electrons and holes, *G* is the generation rate and $D_{n/p}$ is the diffusion coefficient.

Extraction of drift-diffusion models parameters requires different measurements to get accurate results by fitting the experimental results to the model. As the model has large number of parameters, it is more convenient to insert initial values acquired by extraction from conventional models in order to achieve fast convergence. In such case effect of different parameters from conventional models and the drift diffusion model should be identified and the effect of experimental techniques on these parameters is required. From the conventional models series and shunt resistance can be extracted and used to tune the fitting method of the drift-diffusion model. The Rs and $R_{s}h$ can give insights about the microscopic parameters of the device as there is a relation between them. The following section summarizes some experimental work that affects the parasitic resistances.

### 2.4. Series and Shunt Resistances

Series resistance is known as the internal resistance of the device and it has several components affecting its value such as the interfacial connection, active layer resistance, electrode resistances and connect and interconnect resistances. It has a crucial role in determining the solar cell efficiency. It is reported that the resistance of the active layer is inversely related to the mobility of charge carriers. This means high mobility is recommended for better performance although the organic devices exhibit low mobility. Therefore, the series resistance of organic devices is larger than inorganic counterparts. In the numerical extraction techniques, relatively large initial values for $R_{s}$ should be selected for fast convergence. $R_{s}$ was found to significantly affects the FF with minor effect on the cell efficiency for P3HT:PCBM active blend [85]. The same effect was noticed for a blend of MDMO-PPV:PCBM. It was found also the insertion of PEDOT:PSS layer increases the contact component of the series resistance significantly, resulting in a lower fill factor while the thickness of the active layer determines the bulk component [86]. A correlation between device geometry and the series resistance is represented in [87]. The distance between the ITO electrode and the active blend named ITO-bridge was found to increase the device series resistance. Another parameter that affects the series resistance is the device area. In pentacene/C60 devices it was found that, increasing the device area increases the series resistance [88]. The same effect was reported for different organic blends such as P3HT:PCBM and PTB7:PC71BM [89,90]. The increase in the resistance is attributed to the increase in resistance of the conductive electrodes compared to other components. Use of high conductive electrodes is essential for achieving lower resistance. As there a relation between parasitic resistances, carrier mobility and recombination, it is important to mention the effect of mobility on device performance and some experimental methods to measure their values. The following section introduces the effect of charge carrier mobility in combination with recombination in organic solar cells based on different active materials.

## 3. Charge Carrier Mobilities

Charge carrier’s mobility has a strong effect on the performance of the solar cell. The low values of mobilities is known to limit the cell’s short circuit current and the fill factors [91,92]. Also, high mobilities has a competing effect between carrier extraction and recombination [93,94]. At high mobility, the power conversion efficiency is limited by the balance between the dark carrier recombination at open circuit conditions and the photocarrier recombination at short circuit conditions [95]. Not only the value of mobilities is the determining factor for performance but also the balance between the mobilities of holes and electrons. Both fast and slow charges play an important role in determining the performance and the type of the fast and the slow carrier as well, due to the accumulation of fast carrier at the corresponding electrode. In case of hole mobility is larger than the electron mobility a small reduction in *I* is observed [96,97].

Several experimental studies had been done to control charge carrier mobility to improve the solar cell performance. In bulk heterojunction blends the ratio of the donor to the acceptor determine the value of the mobility so, a balance between hole and electron mobility could be achieved by tuning the blend ratio. This ratio also determines the value of mobility as low ratio of acceptor to donor is reported to enhance the mobility [98]. A balanced mobilities had been achieved for P3HT:PCBM blend at ratio of (1:0.3) [99]. In addition to weight ratio mobility could be improved by means of additive materials, thermal annealing, solvent annealing, high boiling point solvents and use of co-solvents. The use of polymer additive poly (N-(4 -(9,9-dioctyl-fluoren-2-yl) phenyl)-N, N0, N0 triphenyl-l,4-phenylenediamine) (PFLAM) to the active blend of P3HT:PCBM improved the hole mobility of the device leading to an increase in the device efficiency by 34% compared to the blend without the additive material [100]. THC8, PCBTDPP, P3HT-b-PS were reported to enhance the hole mobility as well for the same active blend [101,102]. Studies on the effect of thermal annealing on charge carrier mobility showed that the mobility improves with annealed samples compared to non-annealed cells, although the improvement is not always in the direction of increasing temperature. The mobility of an annealed sample of P3HT:PCBM at 140 °C is higher than the mobility of the of the same device annealed at higher temperatures [103]. The same annealing temperature was reported to be the optimal for the same material blend in [104]. Solvent annealing has been observed to have a positive effect on improving the mobility. In a study on a blend of benzothiadiazole based molecule and PC_71_BM as active layer annealed by carbon disulphide an improvement in hole mobility and the power conversion efficiency is achieved [105]. A small molecule device based on BIT4FSe:PC_71_BM annealed by Ch_2_Cl_2_ showed a significant increase in hole mobility and a slight increase in electron mobility [106].

### 3.1. Experimental Techniques for Measuring Charge Carriers Mobility

The following subsections introduces some commonly used experimental methods to measure charge carriers mobility such as charge extraction by increasing voltage, time of flight technique and space charge limited current.

#### 3.1.1. Charge Extraction by Linearly Increasing Voltage

**Figure 4:**
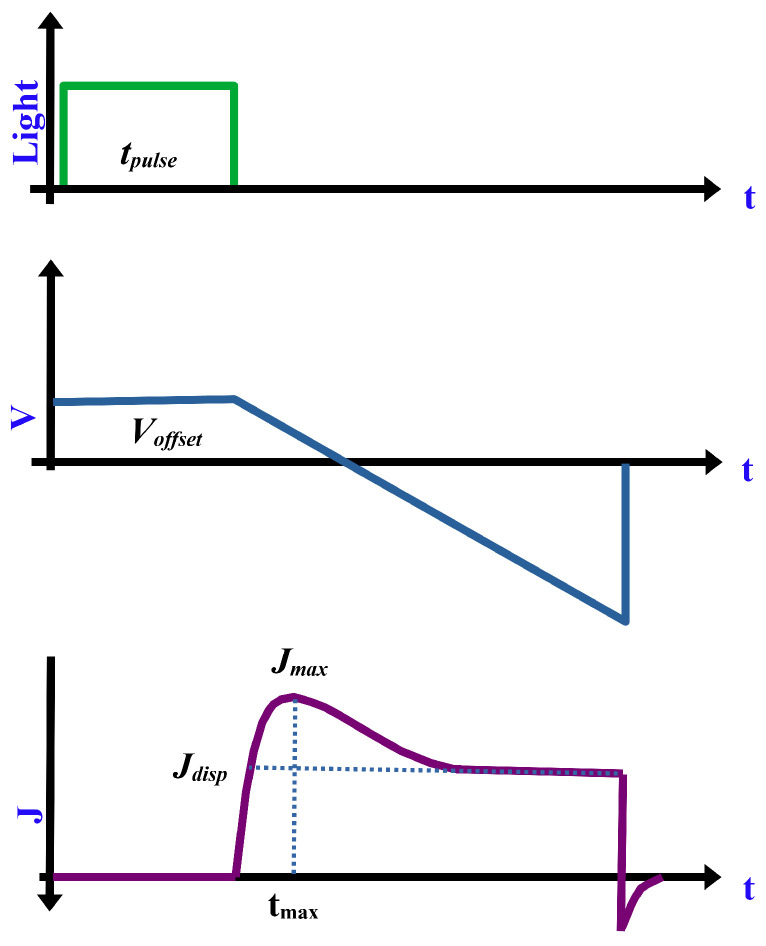
Graphical explanation of photo-CELIV technique [110].

One of the most common techniques that are widely used to measure charge carrier mobility in thin film devices is charge extraction by linearly increasing voltage (CELIV). The technique is based on the collection of photogenerated charges generated by short laser pulse. A voltage ramp in the reverse direction is used to collect the charges leading to a ramp in the displacement current density j(0). The time $t_{max}$ needed by the transient current to reach its peak is used to estimate the mobility in the device through the simple analytical formula given by (15) [107,108,109].

(15) $$\mu =\frac{2\;d^{2}}{3\;A\;t_{max}^{2}}\;\frac{1}{1+0.36\;\frac{\Delta j}{j(0)}}$$

where μ is the mobility of fast carriers, *d* is active layer thickness, *A* is the ramp rate of the applied voltage, Δj is the difference between current at $t_{m}ax$ and j(0). This technique is known as photo-CELIV due to the application of light pulse. A graphical explanation of the technique is given in Figure 4.

**Figure 5:**
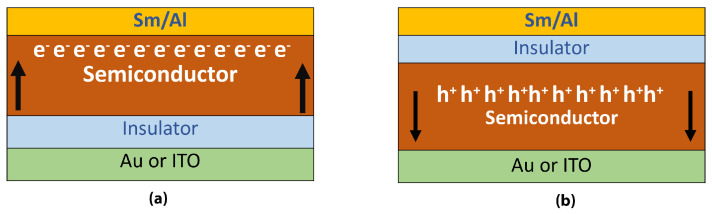
Structure of the device for (**a**) electron selection (**b**) hole selection [111].

A method to measure mobility of each charge carrier is MIS-CELIV which stands for metal-insulated-semiconductor structure. In MIS-CELIV technique injected charge carriers are being extracted instead of photogenerated carriers in traditional photo-CELIV method. The material of interest should be a part of certain structure that consists of two metal electrodes, one of them is connected to an insulating material that is deposited on top or bottom of the semiconductor material according to the type of carrier to be studied. The role of the insulator material is to prevent injection of certain carrier (hole/electron) according to the position of the insulator. To inject holes the insulator must be adjacent to (Sm/Al) electrode. For electron injection a direct connection to (Au/ITO) is necessary. Figure 5, represents the structure used in this method [111,112,113].

#### 3.1.2. Time of Flight Technique

**Figure 6:**
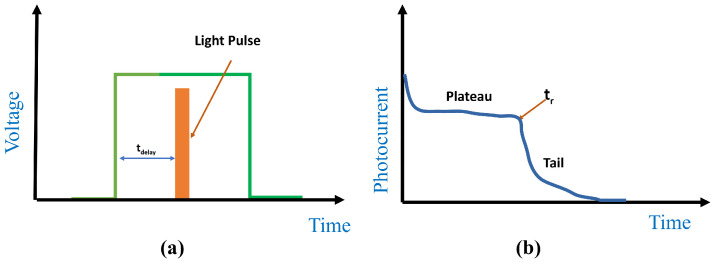
Time of flight technique (**a**) represents the inputs (**b**) represents the outputs [114].

Another technique to measure mobility is time of flight method (TOF). In this method the thin film is placed between two electrodes with at least one of them being transparent. A light pulse is applied through the transparent electrode to generate pairs of charge carriers. The device is connected to external voltage source with its polarity determined by the type of carriers under investigation. The sample is exposed to a light pulse after the application of the voltage. Both type of carriers is photogenerated after the exposure to light pulse which indicated by the spike in Figure 6b [114,115,116]. The decay from the spike to reach the plateau region is due to recombination of one of the carriers at the electrode. The decay from the plateau to reach zero value indicates the recombination of the carrier of interest at the other electrode. The mobility of the carrier can be determined using the following relation.

(16) $$\mu =\frac{d^{2}}{V\;\tau }$$

where *d* is the film thickness, *V* is the applied voltage and τ is the carrier transient time. The experimental setup needed for the two techniques is almost identical in terms of needed devices and instruments. A function generator, digital oscilloscope, and a pulsed light source. Controlling the timing of applied light pulse is important in both techniques and it could be achieved by a programmed microcontroller to achieve synchronization between ramped voltage and pulsed light in case of CELIV and the delay time in case of TOF. CELIV technique is widely used instead of TOF method as the later requires relatively thick films which is not the common case in organic devices as the thickness of active layer is carefully selected for performance optimization [117].

#### 3.1.3. Space Charge Limited Current Model (SCLC)

Space charge limited model is considered and acceptable model to describe charge transport parameters of organic semiconductors such as free carriers density, trap density and effective carriers mobility.Due to the low mobility of charge carriers in organic materials, the injected carriers by external potential will create space charge region inside the device which limits the current. That’s why the current represented by (17) is called space charge limited current (SCLC) [118,119].

(17) $$J=\frac{9}{8}\;\epsilon \;\epsilon_{0}\;\mu \;\frac{V^{2}}{L^{3}}$$

where ϵ is permittivity of the organic material, $\epsilon_{0}$ permittivity of free space, μ is the free carrier mobility, *L* is the device length and *V* is the applied voltage. This model can account for traps also by including θ as the trap factor in Equation (17) that is dependant on the density of free and trapped carriers. The SCLC after inserting θ is given by (18)

(18) $$J=\frac{9}{8}\;\epsilon \;\epsilon_{0}\;\mu \;\theta \;\frac{V^{2}}{L^{3}}$$

## 4. Recombination in Organic Solar Cells

Due to the low dielectric constant of organic semiconductor, the photovoltaic process is different from inorganic materials. In organic devices, the absorbed light generates excited charge carriers (electron-hole) bounded with attractive forces. The exciton then travels to donor/acceptor interface where it separates to free holes and electrons. Electrons transfer to the cathode through the acceptor material and holes move to the anode through the donor domain due to the internal electric field created by the difference in electrodes work functions. Each step in the process exhibits some sort of losses that decreases the device PCE [120,121].

Geminate recombination is one of the mechanisms that contributes to the losses. It is considered a monomolecular process in which excited electron-hole pair generated by the same photon recombine together before dissociation into free charges. Recombination could occur after exciton generation and before moving to the interface or at the interface. Non-geminate recombination occurs after separation of two charge carriers generated by different photons. Non-geminate recombination has three types named monomolecular, bimolecular and trimolecular [122,123].

The most dominant type is the bimolecular recombination, and it has a second order dependance on the charge carrier’s density. It was found that geminate and non-geminate recombination are affected by several factors, and they could be minimized by better tuning these factors. By means of transient absorption spectroscopy, the effect of existence and location of side chains on a family of T8TBT with PCBM had been investigated, revealing that the side chains strongly affect the recombination in the device [124]. Side chain substitution of alkoxy group increases the geminate and non-geminate recombination in PBDT[2H]T:ITIC blend while thienyl-substituted and fluorinated system based on PBD polymers experienced lower recombination and high fill factor [125]. Other factors were found to influence he recombination and ways to minimize them was developed. Proper acceptor structure and HOMO offset level reduces geminate recombination through enhancement of hole extraction [126]. In a system of PBDB-T:O-IDTBR, the ratio of crystallization between donor and acceptor significantly affects recombination losses in the device, blend with large variance in crystallinity suffers from high non-geminate recombination [127]. Table 2, represent some techniques that can be deployed to control recombination in different blend systems.

### 4.1. Experimental Techniques for Recombination Estimation

There are several experimental techniques to estimate recombination in organic electronics, photo-CELIV introduced in the previous section can be used for determining the lifetime of the induced carrier. From the the lifetime, the recombination coefficient can be calculated.

#### 4.1.1. Transient Photovoltage (TPV)

Transient photovoltage is a technique that belongs to the small perturbation category of experimental techniques that can be used do measure carriers lifetime. This technique is used as it mimics the steady state operating conditions of solar cells in which the device is held at open circuit condition at steady illumination. The change induced in the $V_{oc}$ by small light pulse. The decay of the induced voltage ΔV above the $V_{oc}$ is used to measure the life time (τ) given by (19) [135].

(19) $$\tau =\tau_{0}\;exp\left(\frac{q\;V_{oc}}{\upsilon \;K_{B}\;T}\right)$$

where υ is a slope factor that is dependent on the order of recombination dynamics and $\tau_{0}$ is a voltage independent prefactor.

#### 4.1.2. Transient Absorption Spectroscopy (TAS)

A transient optical technique that is widely used to measure carrier’s lifetime is the transient absorption spectroscopy (TAS). In this technique two light pulses with a time delay between them are used to measure the absorption, the first is a pump pulse which induces the charge carriers to the excitation state. The second pulse, is a weak one called the probe pulse. The process of measurement is repetitive and it must be done for different time delays. From the difference in absorption between the case in which the pump pulse exists and the other case without applying it, the change in absorption due to excitation can be determined. After repeating the process for different time delays between the two pulses, the relation between the change in the probe pulse energy and the delay time represents the sample kinetics from which lifetime can be determined [136].

#### 4.1.3. Impedance Spectroscopy (IS)

Impedance spectroscopy belongs to the small perturbation techniques for measuring carrier dynamics. In [137] IS is used to estimate recombination kinetics of a bulk heterojunction organic solar cell with P3HT:PCBM as active layer. In this study the lifetime of electron is represented in terms of internal recombination resistance ($R_{rec}$) and the capacitance ($C_{\mu }^{\left(n\right)}$). Although, the impedance spectroscopy technique is simple in its application but its data needs proper interpretation.

## 5. Parameters Extraction and Its Significance to the Field

In this section extraction of organic solar cell parameters based on P3HT:PCBM active material is represented in Table 3. The examples represented in this section are selected to reflect the importance of parameters extractions step for studying organic solar cells. Firstly, the extraction steps are explained briefly followed by interpretation of the extracted parameters. The extraction procedure starts by extracting the parasitic resistance from the single diode model following the method in [42]. The extraction of $R_{s}$ and $R_{sh}$ can be done using any method listed in Table 1 or the summarized methods in Section 2. Step 2, brings the extracted parasitics from step 1 as input to the drift-diffusion model for extraction of rest of the parameters. The extracted values in Table 3 from [99,138] are based on the J−V measurements. Relying only on current-voltage measurements for fitting to the drift-diffusion model will take time to converge and needs tuning of parameters to minimize the error quickly. It is recommended to include additional measurements such as that mentioned in Section 3 for fast convergence and accurate results.

**Figure 7:**
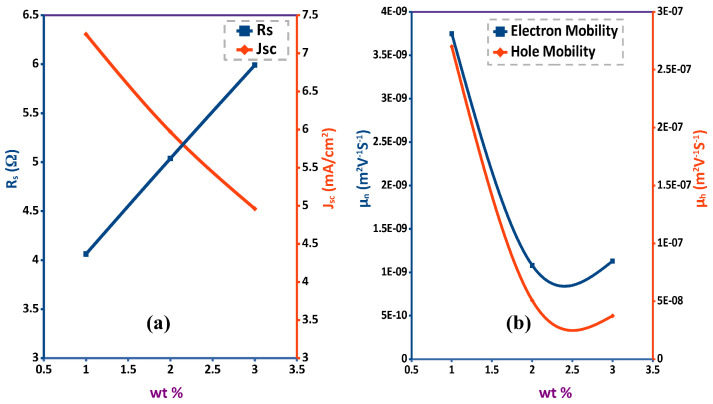
Effect of concentration on: (**a**) series resistance ($R_{s}$) and short circuit current density ($J_{sc}$) (**b**) electron mobility ($\mu_{e}$) and hole mobility ($\mu_{h}$).

Authors of [138] used AFM, FE-TEM and XRD as qualitative techniques to study the effect of changing the concentration of P3HT:PCBM active blend in chlorobenzene. The main focus of the study was to report on the phase separation and morphology of the active layer. It is one of the methods used to study the feasibility of preparing a device according to various schemes. They also performed J−V measurements to study the concentration effect on $V_{oc},J_{sc},FF$ and PCE. Parameters extraction could add more value to such type of work as it can relate the parameter under investigation by means of qualitative measurements such AFM, FE-TEM and XRD to the microscopic parameters such as series resistance, shunt resistance, hole mobility and electron mobility of the device without the need to measure them directly saving time and cost. In Figure 7 extracted ($R_{s}$), ($\mu_{e}$) and ($\mu_{h}$) are plotted against concentration. More information can be obtained by extracting device parameters and doing extra analysis on them. The two steps extraction method based on J−V curve fitting and drift-diffusion model can be used as a compensation for missing experimental measurements to get approximate values of device parameters. In case of available transient measurements such as CELIV the results can be fitted directly to the drift-diffusion model to get the device parameters as done by MacKenzie [107]. It is important to mention that extracted parameters are not the exact values of the device but they offer an alternative solution to study the solar cell quantitatively with minimum number of experimental setups.

## 6. Summary

In this work, the effect of different materials, preparation conditions, device structure, and experimental techniques (Table 4) for studying organic solar cells were introduced in combination with several analytical, numerical, and optimization methods for extracting device parameters for further investigation and performance enhancement. Explanation of some experimental techniques compared to other methods reported was presented as well. Fitting the experimental data from literature was made to extract the parasitic parameters from a single diode model, and then other parameters such as hole mobility and electron mobility were extracted. Methods for parameters extraction based on the double diode model were introduced to be an easily accessible resource for scientists and engineers when the computational power is not necessary compared to the accuracy of the extraction. Several parameters can be extracted from diode and drift-diffusion models, such as ideality and recombination time constant, charge density, and trapped carriers. Researchers can use the work in this review to get insights about their work with minimum resources as a sort of qualitative assessment to their experimental findings so the resources can be invested in a more optimized version of the device under investigation.

## Figures and Tables

**Table 1:** List of solar cell models for parameter extraction based on single and double diode models.

Extracted Parameters	Diode Model	Extraction Method	References
$n,R_{sh},R_{s},I_{ph},I_{D}$	Single	Analytical	[66]
$n,R_{sh},R_{s},I_{ph},I_{D}$	Single	Numerical	[67,68]
$n,R_{sh},R_{s},I_{ph},I_{D}$	Single	Hybrid	[69]
$n,R_{sh},R_{s},I_{ph},I_{D}$	Single	Optimization	[70,71,72,73,74,75]
$n,R_{sh},R_{s}$	Single	Numerical	[76]
$R_{sh},R_{s}$	Single	Analytical	[77]
$n_{1},n_{2},R_{sh},R_{s},I_{ph},I_{D1},I_{D2}$	Double	Numerical	[78]
$n_{1},n_{2},R_{sh},R_{s},I_{ph},I_{D1},I_{D2}$	Double	Hybrid	[79]
$R_{sh},R_{s},I_{ph},I_{D1},I_{D2}$	Double	Numerical	[80]
$n_{1},n_{2},R_{sh},R_{s},I_{ph},I_{D1},I_{D2}$	Double	Optimization	[70,71,72,73,81,82,83,84]

**Table 2:** Effect of different preparation techniques on recombination in different organic solar cells.

Technique	Effect	Active Blend	Reference
Addition of graphene oxide nanosheets	Reduces non-geminate recombination	PTB7:PC_71_BM	[128]
Dichloromethane solvent vapor annealing	Reduces geminate Recombination	BIT-4F:PC_71_BM	[129]
Thermal Annealing	Reduces geminate Recombination	TQ1:N2200	[130]
BTR Small molecule donor	Reduces non-geminate recombination	PCE-10:PC_71_BM	[131]
Donor engineering	Reduces geminate recombination	M0-M3:PC_70_BM	[132]
High molecular weights donor	Reduces non-geminate recombination	PTB7-Th:IDTBR	[133]
Co-solvent DIO (1,8 Diiodooctane)	Reduces geminate recombination	PTB7:PC_71_BM	[134]

**Table 3:** Extracted parameters of different organic solar cells at different conditions.

$R_{s}$(Ω)	$R_{\mathrm{sh}}$(Ω/m^2^)	$\mu_{e}$	$\mu_{h}$		Effect of	Reference
4.0625	$22.25\times 10^{4}$	$3.75\times 10^{-9}$	$2.7\times 10^{-7}$	1wt%		
5.0384	$22.72\times 10^{4}$	$1.08\times 10^{-9}$	$5.07\times 10^{-8}$	2wt%	P3HT:PCBM concentration in solvent	[138]
5.9908	$22.71\times 10^{4}$	$1.13\times 10^{-9}$	$3.74\times 10^{-8}$	3wt%		
5.0501	$39.98\times 10^{4}$	$2.37\times 10^{-8}$	$2.89\times 10^{-9}$	1:0.4		
5.2213	$39.98\times 10^{4}$	$4.22\times 10^{-8}$	$1.79\times 10^{-9}$	1:0.3	P3HT:PCBM ratio	[99]
9.3513	$39.99\times 10^{4}$	$1.047\times 10^{-7}$	$2.56\times 10^{-10}$	1:0.2		
54.1617	$49.51\times 10^{4}$	$1.31\times 10^{-7}$	$2.13\times 10^{-10}$	1:0.1		
19.5	$1.9\times 10^{5}$	$2.48\times 10^{-7}$	$2.48\times 10^{-7}$		Extraction at single condition	[107]

**Table 4:** Experimental techniques for measuring mobility and carrier lifetime.

Technique	Parameter	Reference
photo-CELIV	Mobility of charge carriers	[111]
MIS-CELIV	Mobility of each charge carriers	[112]
Time of flight (TOF)	Mobility of charge carriers	[116]
Space charge limited current (SCLC)	Mobility of charge carriers	[119]
Transient photovpltage (TPV)	Carriers lifetime/Recombination	[135]
Transient absorption spectroscopy (TAS)	Carriers lifetime/Recombination	[136]
Impedance spectroscopy (IS)	Carriers lifetime/Recombination	[137]

## Data Availability

This study did not report any data.
